# Interactions between Anaerobic Fungi and Methanogens in the Rumen and Their Biotechnological Potential in Biogas Production from Lignocellulosic Materials

**DOI:** 10.3390/microorganisms9010190

**Published:** 2021-01-17

**Authors:** Yuqi Li, Zhenxiang Meng, Yao Xu, Qicheng Shi, Yuping Ma, Min Aung, Yanfen Cheng, Weiyun Zhu

**Affiliations:** 1 Laboratory of Gastrointestinal Microbiology, National Center for International Research on Animal Gut Nutrition, Nanjing Agricultural University, Nanjing 210095, China; 2018105039@njau.edu.cn (Y.L.); 2019205023@njau.edu.cn (Z.M.); 2019105043@stu.njau.edu.cn (Y.X.); 2017105038@njau.edu.cn (Q.S.); 2018105071@njau.edu.cn (Y.M.); minaung.uvs@gmail.com (M.A.); zhuweiyun@njau.edu.cn (W.Z.); 2 Department of Animal Nutrition, University of Veterinary Science, Nay Pyi Taw 15013, Myanmar

**Keywords:** anaerobic fungi, methanogens, lignocellulose, methane

## Abstract

Anaerobic fungi in the digestive tract of herbivores are one of the critical types of fiber-degrading microorganisms present in the rumen. They degrade lignocellulosic materials using unique rhizoid structures and a diverse range of fiber-degrading enzymes, producing metabolic products such as H_2_/CO_2_, formate, lactate, acetate, and ethanol. Methanogens in the rumen utilize some of these products (e.g., H_2_ and formate) to produce methane. An investigation of the interactions between anaerobic fungi and methanogens is helpful as it provides valuable insight into the microbial interactions within the rumen. During the last few decades, research has demonstrated that anaerobic fungi stimulate the growth of methanogens and maintain methanogenic diversity. Meanwhile, methanogens increase the fiber-degrading capability of anaerobic fungi and stimulate metabolic pathways in the fungal hydrogenosome. The ability of co-cultures of anaerobic fungi and methanogens to degrade fiber and produce methane could potentially be a valuable method for the degradation of lignocellulosic materials and methane production.

## 1. Introduction

The rumen is a major compartment of ruminant stomachs and is well established as a natural and efficient system for crude fiber fermentation [1]. Rumen microbes anaerobically ferment complex lignocellulosic plant materials which cannot be directly utilized by a host, into monomers which are further degraded into different microbial end-products, including volatile fatty acids (VFAs), hydrogen (H_2_), carbon dioxide (CO_2_), methane (CH_4_), and other fermentation products necessary for essential metabolic pathways [2]. The complex rumen microbiome network is dominated by bacteria, archaea, protozoa, and anaerobic fungi [3]. It has been previously proposed that bacteria and protozoa are primarily responsible for the degradation of plant tissues within the rumen, as these microbes are more abundant than anaerobic fungi by orders of magnitude. However, anaerobic fungi belonging to the phylum Neocallimastigomycota have been found to account for up to 70% of plant tissue degradation in both ruminant and non-ruminant herbivores [4]. Methanogenic archaea can produce methane through interspecific H_2_ transfer using the products of anaerobic fungal metabolism, improving the crude fiber degradation capability of anaerobic fungi [5].

The balance of rumen fermentation mainly depends on microbial interactions, such as the associated activity of anaerobic fungi and methanogens. Previous studies have comprehensively clarified the close relationship between anaerobic fungi and methanogens [6,7,8,9]. It has been well established that interspecies H_2_ transfer produces methane and enhances the regeneration of oxidized nucleotides, such as NAD+ and NADP+ [10]. Furthermore, H_2_ transfer also alters anaerobic fungal metabolic pathways (such as the lactate and ethanol pathways), resulting in greater production of acetate, formate, and ATP [11]. Co-cultures have been found to exhibit enhanced anaerobic fungi growth rates, accompanied with increased rates of cellulolytic enzyme activity and dry matter reduction, as well as enhanced rates of methanogenesis [6,7,8,12,13]. In recent years, due to these distinctive traits, many studies have investigated the biogas potential of anaerobic fungi, finding that they can successfully increase methanogen CH_4_ yields in co-cultures [6,7,8,9,13,14,15,16,17]. Furthermore, co-culture mode biogas production can decompose lignocellulosic-rich plant materials without any pretreatment [18].

This review assesses the reported interactions between anaerobic fungi and methanogens, clarifying the roles that anaerobic fungi and methanogens undertake in this syntrophic relationship. Furthermore, the future perspectives and biotechnological potential of anaerobic fungi and methanogen co-cultures in lignocellulose degradation and methane production are discussed.

## 2. Taxonomy, Distribution, Metabolism, and Fiber Degrading Enzymes of Anaerobic Fungi

The rumen is the most efficient natural system known for the degradation of plant celluloses and it contains a large number of microorganisms, including anaerobic fungi, archaea, protozoa, and bacteria. The microbial community in the rumen is capable of degrading crude fiber that cannot be digested by a host animal, maintaining high metabolic performance and the health of ruminants. Anaerobic fungi are one of the first microorganisms in the rumen to colonize the fibrous tissue of plants, efficiently degrading the components of plant cell walls [19]. Before anaerobic fungi were confirmed to exist, the scientific community generally believed that fungi were obligate aerobic microorganisms. Therefore, when the zoospores of rumen anaerobic fungi were first discovered, they were initially defined as “protozoa” [20] and, subsequently, classified as phycomycetes [21] and chytridiomycetes [22], until Orpin et al. [21] showed that these fungi were strictly anaerobic. Since their discovery, an increasing number of anaerobic fungi have been isolated and cultured. In 2007, anaerobic fungi were classified as an independent phylum called Neocallimastigomycota [23]. To date, the following eighteen genera of anaerobic fungi have been identified (Table 1) according to their growth type, rhizoidal morphology, and number of zoospore flagellum [24]: *Neocallimastix, Caecomyces, Orpinomyces, Piromyces, Anaeromyces, Cyllamyces, Buwchfawromyces, Oontomyces, Pecoramyces, Feramyces, Liebetanzomyces, Agriosomyces, Aklioshbomyces, Capellomyces, Ghazallomyces, Joblinomyces, Khoyollomyces,* and *Tahromyces.* As shown in Table 1, it has been nearly four decades since the first anaerobic fungi were identified and since then, most anaerobic fungi have been isolated from ruminant feces. The main reason for this is that the rumen is in an anaerobic environment with the appropriate pH for ingesting plant tissues that can remain within the digestive tract for a long duration [25]. These conditions result in the rumen containing a high abundance of anaerobic fungi (about 10^6^/g wet ruminal content) [26]. Almost all genera of anaerobic fungi can be isolated from feces, which indicates that anaerobic fungi have an oxygen-resistant structure [27], causing them to become dormant under certain conditions, and thereby resist adverse environmental effects. The methods for isolating anaerobic fungi from animal feces have been well established and widely applied, greatly improving our understanding of anaerobic fungi and showing that the distribution of anaerobic fungi in nature is far more extensive than previously known.

After anaerobic fungi were found in the rumen of ruminants [20], they were found to be involved with fermentation in the posterior intestine of herbivorous mammals [39], the foregut of fermentation-type ruminants [40] and non-ruminants [41], large herbivorous rodents [42], and herbivorous reptiles [43]. Moreover, the habitats where anaerobic fungi exist have also been extended from the digestive tract [20,21] and feces [40,44], to the soil [45] and the deep sea [46]. Although the presence of anaerobic fungi has been detected via biomolecular analysis in these environments in vitro, to date there have been no reports of anaerobic fungi isolated from environments other than the intestine. This implies that there may be many undiscovered anaerobic fungi present throughout nature and the diversity of anaerobic fungi is far greater than that capable of being obtained by isolation and culturing. Factors such as host intestinal type, region, and diet all affect the diversity of anaerobic fungi [25]. The abundance of anaerobic fungi is mainly affected by the composition of the host’s diet [7], with an increase in the number of anaerobic fungi when the host is fed a high-fiber diet [47,48]. The ability to isolate anaerobic fungi depends on the similarity between the medium and its habitat; a higher diversity of anaerobic fungi is isolated with a higher level of similarity, more closely reflecting the diversity in their original habitat. However, it is difficult to maintain a strict anaerobic environment in vitro. As a result, researchers have utilized methods that do not rely on culture methods to assess the diversity of anaerobic fungi. Edwards et al. [49] studied the diversity of anaerobic fungi colonized on plant fiber tissue by amplified ribosomal intergenic spacer analysis (ARISA), identifying two multi-centered genera, *Anaeromyces* and *Orpinomyces*, within 30 min. Cheng et al. [6] used ARISA to show that different passage frequencies have a high impact on the diversity of anaerobic fungi. However, the results of ARISA are based on the ITS1 region length polymorphism and because of the complexity of anaerobic fungi in samples, this method did not adequately identify the anaerobic fungi in samples. Nicholson et al. [50] used denaturing gradinent electrophoresis (DGGE) and Spreadex gel combined with gel sequencing to study the diversity of anaerobic fungi in wild-type and non-wild-type herbivore feces. However, this method did not effectively analyze the proportion of identified anaerobic fungi. With the rapid development of sequencing technology, an increasing number of studies have applied next-generation sequencing technology to assess the diversity of anaerobic fungi. Liggenstoffer et al. [43] used high-throughput sequencing technology to show that *Piromyces* is the most widely distributed and abundant anaerobic fungal genus in the studied herbivore feces samples, with the host species having a great influence on the abundance of anaerobic fungi. Kittelmann et al. [51] used high-throughput sequencing technology to show that the dominant anaerobic fungi in the rumen of ruminants are *Neocallimastix* and *Piromyces*. Mao et al. [26] used high-throughput sequencing technology to study the diversity of anaerobic fungi in the rumen of goats, showing that the proportion of roughage in the diet significantly affected the community diversity and abundance of anaerobic fungi. Although high-throughput techniques can yield large amounts of data, sequencing lengths are limited, and this method is not convenient for phylogenetic analysis and comparisons with individual sequences. Overall, the diversity of anaerobic fungi and the dominant flora have been shown to be affected by factors such as host species, dietary type, and regional differences in different reports.

**Figure 1:**
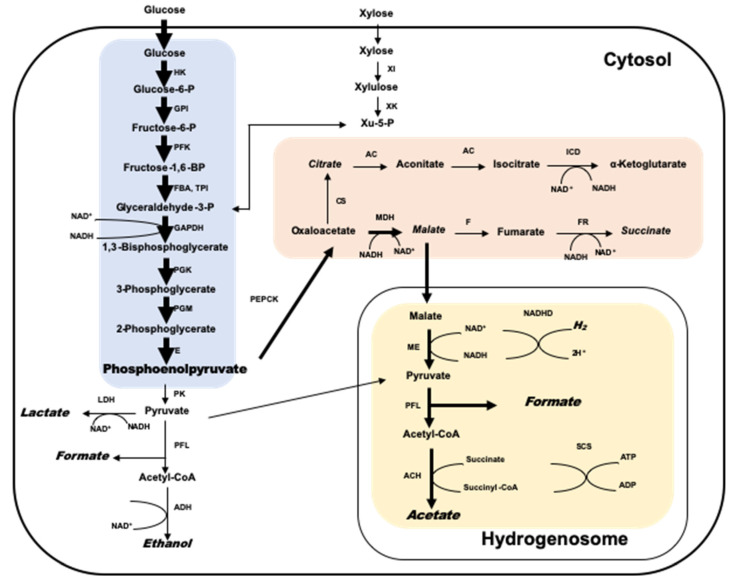
Glucose and xylose metabolism by anaerobic fungi. The main path is indicated by bold arrows. The proposed metabolites are indicated in italics. AC, aconitase; ACH, acetyl-CoA hydrolase; ADH, alkoholdehydrogenase; CS, citrate synthase; E, enolase; FBA, fructosebisphosphate aldolase; F, fumarase; FR, fumarate reductase; GAPDH, glyceraldehyde-3-phosphate dehydrogenase; GPI, glucose-6-phosphate isomerase; HK, hexokinase; ICD, isocitrate dehydrogenase; LDH, lactatedehydrogenase; MDH, malate dehydrogenase; ME, malic enzyme; NADHD, NADH dehydrogenase; SCS, succinyl-CoA synthetase; TPI, triosephosphate isomerase; XK, xylulokinase; XI, xylose isomerase; PFK, phosphofructokinase; PFL, pyruvate formate lyase; PGK, 3-phosphoglycerate kinase; PGM, phosphoglucomutase; PK, pyruvate kinase; PEPCK, phosphoenolpyruvate carboxykinase.

Anaerobic fungi can fertilize a wide range of substrates, including complex structural polysaccharides, soluble sugars, and storage polysaccharides [23], using a series of enzymes to degrade plant cell walls into soluble sugars that can be secreted [52,53]. These sugars are metabolized by intracellular enzymes into H_2_, CO_2_, formate, acetate, ethanol, and succinic acid after entering the cytosol of anaerobic fungi [13,54]. Research has shown that the metabolites formed by anaerobic fungi using different carbon sources remain the same (Figure 1), mainly formate, acetate, ethanol, lactate, CO_2_, and H_2_, while different substrates produce different proportions of metabolites [55]. Anaerobic fungi undergo mixed acid fermentation to produce formate, acetate, lactate, ethanol, succinic acid, H_2_, and CO_2_, and as they do not contain mitochondria, and they use a hydrogenosome to produce ATP [55]. In addition, the hydrogenosome can produce H_2_, CO_2_, acetate, and formate [56]. Two key enzymes in the anaerobic fungi metabolic pathway are pyruvate formate lyase (PEL) and alcohol dehydrogenase E (ADHE), which is significantly different from other eukaryotic microorganisms [54]. In addition, anaerobic fungi secrete an abundance of carbohydrate active enzymes (CAZymes) during the degradation of lignocellulosic materials.

**Figure 2:**
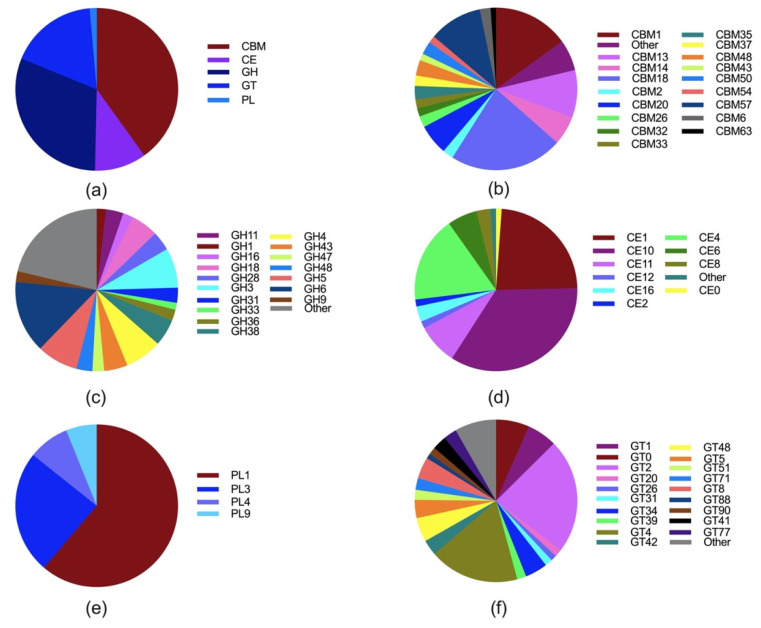
The CAZymes in anaerobic fungal strain *Pecoramyces* sp. F1. The identified complement of (**a**) CAZymes; (**b**) Carbohydrate binding modules (CBM); (**c**) glycoside hydrolases (GH); (**d**) Carbohydrate esterases (CE); (**e**) Polysaccharide lyases (PL); (**f**) Glycosyl transferases (GT).

In recent years, transcriptome analysis has shown that anaerobic fungi can express an extensive array of transcripts to encode a range of CAZymes [57]. To further elucidate the digestive mechanisms of lignocellulosic materials by anaerobic fungi, a detailed analysis and comparison of the complement of CAZymes expressed in *Pecoramyces* sp. F1 was undertaken (Figure 2a). *Pecoramyces* sp. F1 expressed a large number of carbohydrate binding modules (CBMs), as well as various genes encoding proteins containing CAZyme modules (glycoside hydrolases, carbohydrate esterases, glycosyltransferases, and polysaccharide lyases). In *Pecoramyces* sp. F1, 40% of the contigs encoding CAZymes had known CBMs, with the commonly observed CBMs being CBM18, CBM1, and CBM57 (Figure 2b). The most diverse group of CAZymes in anaerobic fungi was the glycoside hydrolase (GH), with approximately 30% of the complement of CAZymes expressed. In the ranked order of highest to lowest abundance, the 10 most abundant families in anaerobic fungi were: GH6, GH5, GH3, GH4, GH18, GH38, GH43, GH28, GH11, and GH48 (Figure 2c). The three most abundant carbohydrate esterase (CE) families in *Pecoramyces* sp. F1 were CE10, CE1, and CE4 (Figure 2d). *Pecoramyces* sp. F1 also possessed members of four families of putative polysaccharide lyases (PL) (PL1, PL3, PL4, and PL9) which may have activity specific for pectin (Figure 2e). In contrast to the glycosyl hydrolases, the number of identified glycosyl transferases was comparatively small, accounting for only about 17% of the CAZyme complement. The most abundant glycosyltransferases (GT) families were GT2, GT4, GT0, GT1, GT48, and GT8 (Figure 2f), which are involved in various metabolic processes including cell wall biosynthesis, chitin synthesis, and glycosylation [58]. These unique traits enable anaerobic fungi to break down untreated cellulosic material. Recently, an analysis of the lignocellulolytic machinery in the anaerobic fungi *Orpinomyces* sp. strain C1A genome revealed a highly diverse CAZyme range consisting of genes for 357 glycoside hydrolases, 24 polysaccharide lyases, and 92 carbohydrate esterases [59]. To date, most anaerobic fungi have been reported to produce these three enzyme types, which are needed to degrade plant biomass [56]. Using transcriptomic sequencing technology, Wang et al. [60] studied the expression of carbohydrate-related enzymes for fermentation by anaerobic fungi (*Neocallimastix Patriciarum* W5) in the presence of different substrates, identifying 219 different glycoside hydrolases under 25 different GH families. Couger et al. [61] showed that when the anaerobic fungi *Pecoramyces ruminantium* C1A were fermented with different lignocellulosic substrates, there was no significant difference in the expression of CAZymes, suggesting that there could be core genes expressed in anaerobic fungi and this is not affected by variations in the substrate. Although great breakthroughs have been made in transcriptomic research on anaerobic fungi, a large number of anaerobic fungi transcriptomes have not yet been functionally annotated, which seriously hinders the research on CAZymes.

## 3. Taxonomy, Distribution, and Metabolism of Methanogens

Methanogens are strictly anaerobic, methane-producing archaea, which produce methane as the end-product of anaerobic respiration [62]. Methanogens have been found in the gastrointestinal tract of almost all vertebrates and most methane producers have a plant-based diet. Ruminants such as cattle, sheep and goats, and hindgut fermenters such as horses and elephants can produce large amounts of methane. Methane is also released by some animals that eat high protein foods, such as carnivorous crocodiles, giant snakes, and ant-eating species such as tamandua and the aardvark, which can release large amounts of methane from the feces of ingested animals and insects [63]. Although methanogens are widely distributed throughout the natural environment, all methanogens have three common characteristics: firstly, they all produce methane, utilizing all or most of their energy for the production of large amounts of methane. Secondly, they are archaea, belonging to the phylum Euryarchaeota. Lastly, all methanogens are obligate anaerobes, growing only in strictly anaerobic environments [62]. The strict requirement for anaerobic survival has hindered the isolation and culture of methanogens in vitro and the first successful methanogen isolates have been *Methanosarcina Barkeri* and *Methanobacterium formicium* [63]. In recent years, due to the improvement of anaerobic separation technology in combination with advanced identification methods, more methanogenic strains have been identified. Currently, methanogens are classified into the following four taxonomic classes (Table 2): Methanobacteria, Methanococci, Methanomicrobia, and Methanopyri, which are further divided into the following seven orders: Methanobacteriales, Methanococcales, Methanomicrobiales, Methanopyrales, Methanosarcinales, Methanocellales [64], and the newly established order Methanomassiliicoccales (Mmc) [65]. The seven orders of methanogens are divided into 15 families and 35 genera. In the rumen, methanogens have been found to account for less than 1% of the total community of microorganisms, maintaining a cooperative relationship with H_2_ producing microorganisms. The rumen is a complex ecosystem in which the structure of the methanogenic community is not fixed, being affected by various factors such as the animal species, diet, and environment. Statistical analysis has shown that the four most common genera of methanogens in ruminants include *Methanobacterium*, *Methanobrevibacter*, *Methanomicrobium*, and *Methanosarcine* [58].

According to the study by Pierre et al. [66], *Methanobrevibacter* is a dominant methanogen in the digestive tract of herbivores, with many studies investigating the various species of *Methanobrevibacter* present in the rumen. King et al. [67] divided *Methanobrevibacter* into two categories according to their phylogenetic distribution, referred to as the *Methanobrevibacter* of SGMT and RO. In recent years, studies on the distribution of SGMT and RO *Methanobrevibacter* in the alimentary tract of herbivores have shown that although *Methanobrevibacter* is the dominant flora, the proportion of SGMT and RO *Methanobrevibacter* in the alimentary tract of different animals varies significantly. In recent years, many studies have shown that Mmc is the second largest order of methanogens in the rumen, becoming the most dominant group under certain conditions [68]. Huang et al. [69] investigated methanogens in the rumen of yaks and cattle on the Qinghai-Tibet Plateau, and found that the proportion of Mmc in the rumen of yaks and cattle reached 80.9% and 62.9%, respectively. Seedorf et al. [70] studied the structure of methanogens in the rumen of grazing ruminants in New Zealand, with results showing that Mmc accounted for 10.4% of the total methanogen community on average. Similar to anaerobic fungi, methanogens are widely distributed throughout the natural environment. In addition to the ruminant intestinal tract, methanogens exist in wetlands [71], paddy fields [72], fresh water [73] and marine sediments [74], plant rhizospheres [75], underground oil [76] and coal reservoirs [77], anaerobic digesters [78], and other natural and artificial environments. Furthermore, methanogens have been found to survive in extreme environments such as arid deserts [79] and hot springs [80].

The wide distribution of methanogens is closely related to their metabolic characteristics. Methanogens can be divided into the following three groups according to their preferred substrate: hydrogenotrophic (reducing CO_2_, methane and H_2_, or formate), acetoclastic (cleaving carbon-carbon bonds to produce methane from the methyl moiety), and methylotrophic methanogens (producing methane from methyl groups using electrons provided from oxidation). Methanogens are a class of anaerobic microorganisms with a unique evolutionary system, being strictly anaerobic and playing a role in the final steps of metabolism in the rumen. Gram staining of the cell wall of methanogens can be positive or negative, although the cell wall composition is very different from that of bacteria. Bacterial cell walls contain a typical chitosan polymer, while the cell wall of methanogens contains pseudocytoplasm, heteropolysaccharides, or proteins [81] which do not contain cytokinic acid, diaminophenic acid, or teichoic acid, while fat is in the form of glycerol ether instead of glyceride. Furthermore, methanogens cannot utilize complex organic compounds, but can grow in media containing methanol, ammonia, and sulfide. To obtain energy, methanogens can convert CO_2_, H_2_, formate, methanol, acetate, methylamine, and other compounds into methane or methane and CO_2_, being the only known microorganisms to use methane as a final metabolite [82]. In addition, methanogens also contain special coenzymes such as CoM, methylreductase, and F420. Some hydrogenotrophic methanogens also require acetate as a carbon source, although this depends on the type or strain of methanogen. Physiological characteristics of methanogens are highly diverse, ranging from psychrophiles to hyperthermophiles, acidophiles to alkaliphiles, and non-halotolerant to extreme halophilic species, and therefore growth conditions must be adjusted specifically for the respective trait [75]. As a typical hydrogenotrophic methanogen, *Methanobrevibacter* is the dominant archaea in the rumen, resulting in most of the methane produced in the rumen being generated by hydrogenotrophic type methanogens. As the second largest group of methanogens in the rumen, Mmc is a methylotrophic methanogen [76], which mainly uses methyl compounds to generate methane. However, the role and contribution of Mmc in rumen remains unclear and requires further study.

## 4. Interaction of Anaerobic Fungi and Methanogens in the Rumen

To study the metabolic relationship between methanogens and anaerobic fungi in the rumen, Cheng et al. [6] utilized the insensitivity of methanogens and anaerobic fungi to penicillin and streptomycin, establishing a novel mixed co-culture system using methanogens and anaerobic fungi, to assess the influence of different passage frequencies on microflora in the co-culture system. According to the available literature, methanogens and anaerobic fungi can form stable co-culture systems in vitro [83], with analysis showing that the metabolite components of anaerobic fungi are relatively complex, including formate, acetate, lactate, succinic acid, ethanol, H_2_, and CO_2_ [5], among which formate, H, and CO_2_ are the main substrates used by methanogens in the rumen. According to the research of Li et al. [84], *Methanobrevibacter* in the rumen are typically hydrogenotrophic methanogens, which mainly use H_2_ or formate to reduce CO_2_ for the production of methane. In the co-culture system of *Methanobrevibacter* and anaerobic fungi, almost no H_2_ accumulation is observed, while a large amount of H_2_ is accumulated in pure anaerobic fungi cultures, indicating that the anaerobic fungi metabolites H_2_ and formate can be rapidly utilized by methanogens, reducing the feedback inhibition of H_2_ products, promoting the carbon source flow to the hydrogenosome, and enhancing the hydrogenosome metabolic pathway in anaerobic fungi [85]. Therefore, the degradation of lignocellulosic biomass by anaerobic fungi and methane production can be significantly improved by increasing the metabolic capacity of the hydrogenosome. Studies have also found that co-culture of methanogens with anaerobic fungi resulted in the upregulation of multiple CAZyme families in anaerobic fungi, improving their ability to sense and import carbohydrates [86]. The upregulation of CAZymes in co-culture with methanogens provides verification that future efforts to engineer anaerobic intestinal fungi as platforms for biomass degradation and biogas production, are highly likely to benefit from the inclusion of a methanogenic partner, utilizing the synergy between these two organism types. The main reason for the intimate relationship between anaerobic fungi and methanogens may be that methanogens can efficiently utilize the substances generated by anaerobic fungi [8]. Anaerobic fungi metabolites change when methanogens use H_2_ produced by anaerobic fungi, relieving the H_2_ inhibition on the hydrogenosome, and thereby catalyzing more NAD(P)H to produce H_2_. Therefore, more carbohydrates are metabolized by this pathway into the hydrogenosome for the production of acetate and ATP. However, the formation of lactate and ethanol requires the participation of NAD(P)H, therefore, the production of lactate and ethanol is inhibited [87]. There has been little accumulation of formate observed in co-culture experiments, with a decrease in the concentration of lactate, the concentration of acetate has increased [6]. All the above reports utilized an anaerobic fungi medium with no artificial H_2_, CO_2_, or formate added to the fermentation system. Methanogens are unable to grow when anaerobic fungi are inhibited and H_2_ and CO_2_, or formate are added to the system [6], indicating that anaerobic fungi provide energy substances to methanogens and provide essential nutrients for growth. In summary, there is a definite relationship between anaerobic fungi and methanogens, although the mechanism of interaction requires further investigation.

**Figure 3:**
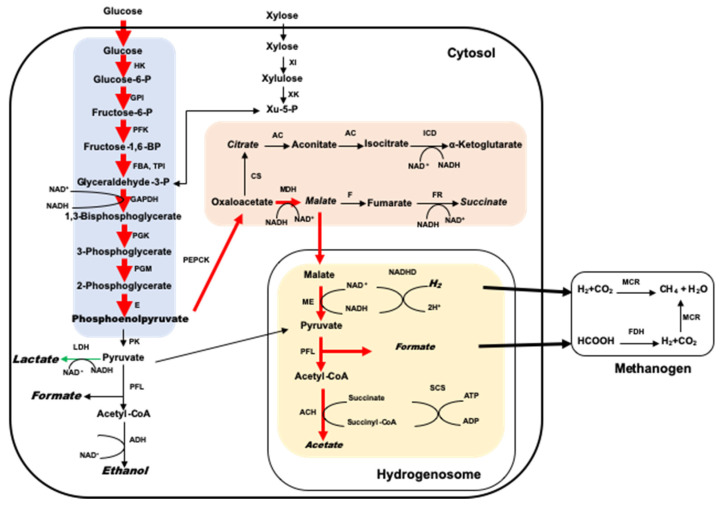
Proposed metabolic pathway for glucose by anaerobic fungi co-cultured with methanogens. The red and green arrows indicate stimulated and inhibited pathways. The proposed metabolites are indicated in italics. AC, aconitase; ACH, acetyl-CoA hydrolase; ADH, alkoholdehydrogenase; CS, citrate synthase; E, enolase; F, fumarase; FBA, fructosebisphosphate aldolase; FDH, formate dehydrogenase; FR, fumarate reductase; GAPDH, glyceraldehyde-3-phosphate dehydrogenase; GPI, glucose-6-phosphate isomerase; HK, hexokinase; ICD, isocitrate dehydrogenase; LDH, lactatedehydrogenase; MCR, methyl coenzyme-M reductase; MDH, malate dehydrogenase; ME, malic enzyme; NADHD, NADH dehydrogenase; SCS, succinyl-CoA synthetase; TPI, triosephosphate isomerase; XK, xylulokinase; XI, xylose isomerase; PFK, phosphofructokinase; PFL, pyruvate formate lyase; PGK, 3-phosphoglycerate kinase; PGM, phosphoglucomutase; PK, pyruvate kinase; PEPCK, phosphoenolpyruvate carboxykinase.

As compared with other co-culture studies, such as some simple co-culture combinations of isolated and purified methanogenic strains and anaerobic fungal strains [12], due to the presence of multiple methanogens and anaerobic fungi in mixed co-culture systems, it is difficult to elucidate the effects of co-culture methanogens on the metabolism of anaerobic fungi or to establish the relevant regulatory mechanisms. The close relationship between co-cultures of single anaerobic fungi with methanogens is reflected by the physical location and rumen metabolism. Bauchop et al. [5] found that anaerobic fungi and methanogens exhibit a direct symbiotic relationship, with almost no accumulation of H_2_ or formate in co-cultured metabolites as compared with pure anaerobic fungi systems, with a reduction in lactate and ethanol and an increase in the concentration of acetate. (Figure 3). In co-culture, methanogens attach to the rhizoid surface of anaerobic fungi [8], with some anaerobic fungi not surviving when methanogens are specifically suppressed by antibiotics. Using the fluorescence in situ hybridization (FISH) technique, Leis et al. [88] found that a large number of methanogens were attached to the sporangium surface of anaerobic fungi. A large number of reports have shown that when methanogens and anaerobic fungi are co-cultured, the degradation of lignocellulosic materials by anaerobic fungi can be significantly improved. The presence of methanogens significantly improves the xylanase activity in anaerobic fungi and improves the utilization of xylose [89]. Some studies have mixed anaerobic fungi with two different methanogens in artificial mixed cultures (using H_2_ and formate or acetate as substrates) and found that these co-cultures resulted in more substrate utilization than simple co-cultures of anaerobic fungi with a single methanogen [12,83]. Co-culture can increase the degradation rate of lignocellulose for two reasons, first, methanogens utilize the metabolites of anaerobic fungi (H_2_ and formate) and, secondly, anaerobic fungi promote the formation of ATP, providing adequate energy to grow without product inhibition. Therefore, the number of cells increases rapidly, and the enzyme yield is also significantly increased.

## 5. The Biotechnological Potential for Biogas Production from Lignocellulosic Materials

A common problem in anaerobic digestion is that the degradation capacity of lignocellulosic materials is limited, which is attributed to the physical structure and the chemical properties of these materials [90]. Preliminary work on biogas production from lignocellulosic materials requires pretreatment; common physical and chemical pretreatment methods are expensive and recycling the acid and alkali after pretreatment also presents a major environmental challenge [91]. Co-culture of anaerobic fungi and methanogens is an essential first step in the degradation of roughage in the rumen, which efficiently and powerfully degrades lignocellulosic materials and efficiently produces methane in an integrated process [92,93]. As compared with the biogas production steps of pretreatment-saccharification-energy production commonly used in industrial applications, the integrated procedure used in co-culture involves simultaneous pretreatment, saccharification, and production steps. This saves time, reduces operational costs, and avoids the production of secondary environmental pollutants, and therefore contributing to sustainable biogas development [94]. Published research indicates that anaerobic fungi degrade fibrous substrates via a series of fiber-degrading enzymes [90], in combination with the penetration of rhizoids [9,52]. Furthermore, syntrophic methanogens can accelerate the growth of anaerobic fungi [13,84] and can enhance the capacity of anaerobic fungi for lignocellulosic digestion [6]. Recent research has shown that an increased complexity of substrates upregulates the expression of CAZymes in anaerobic fungi [95], resulting in co-cultured anaerobic fungi and methanogens being able to degrade more recalcitrant lignocellulosic materials. Overall, these results imply that co-cultures of anaerobic fungi and methanogens have expansive potential for the depolymerization and digestion of complex lignocelluloses. The yield of methane depends on the species of co-culture anaerobic fungi and methanogens, and also depends on the nutritional value of fermentation substrates. By mixing the lignocellulosic materials with other wastes (such as wastewater sludge and chicken manure), the efficiency of methane conversion can be improved [96,97]. A mixed substrate means a better balance of carbon, phosphorus, and nitrogen (C/P/N), and phosphorus is the basic element to promote the optimal growth of methanogens [96]. At the same time, an appropriate ratio of C/N can accelerate the rate of substrate metabolism in fermentation. Some studies have shown that a C/N ratio of about 25:1 results in the highest methane synthesis output [98]. However, more studies also need to focus on mixing different low value substrates to achieve a C/P/N balance.

Various publications have shown the capacity for methane production from various lignocellulosic substrates using co-cultured anaerobic fungi and methanogens (Table 3). Bauchop et al. [5] used co-cultured anaerobic fungi and a rumen H_2_-formate-utilizing methanogen to ferment filter paper strips (cellulose) and obtained 1.78 mmol methane/g substrate after incubation for 3 days. Mountfort et al. [83] poly-cultured one strain of anaerobic fungi with two strains of methanogen and used filter paper, sisal twin fiber, and barley straw leaf strips to produce methane. With sisal twin fiber as a substrate, co-cultures of anaerobic fungi and *Methanobrevibacter* sp. strain RAl could generate 2.1 mmol methane/g substrate in 3 days, while tri-culture of an anaerobic fungi, *Methanobrevibacter* sp. strain RAl and *Methanosarcina barkeri* strain 227, produced 10.1 mmol methane/g substrate in 19 days. Using cellulose as a substrate, co-culture of *Neocallimastix frontalis* and the formate-utilizing methanogen *Methanobacterium formicicum* produced 5.7 mmol methane/g substrate in 7 days [12]. Joblin et al. [99] showed that co-culture of *Neocallimastix frontalis* and *Methanobrevibacter smithii* degraded 30 ± 1% of ryegrass stem to synthesize 10.75 mL methane/g substrate after 6 days. In contrast, using barley straw as a substrate, Cheng et al. [6] used a natural enrichment of anaerobic fungi and methanogens to produce a maximum of 1.75 mmol methane/g substrate in 3 days. Jin et al. [8] reported that two robust co-cultures of anaerobic fungi and methanogens generated approximately 1.6 and 1.8 mmol methane/g bagasse in 4 days. Wei et al. [100] reported that a simple co-culture of *Neocallimastix frontalis* and *Methanobrevibacter ruminantium* isolated from yaks, could produce 3.0 mmol methane/g wheat straw, 3.29 mmol methane/g corn stalk, and 3.15 mmol methane/g rice straw, after 7 days. Recent research utilized a co-culture of anaerobic fungi and methanogens to digest untreated and steam-exploded corn stover and produced 37.1 mL methane/g substrate and 32.2 mL methane/g substrate in 3 days, respectively [18]. Overall, the co-culture of anaerobic fungi and associated methanogens appears to be a highly promising inoculum system for anaerobic digestion and biogas production of lignocelluloses. However, there remain some limitations that must be resolved prior to the practical application of this method, such as the difficulty in isolating efficient co-cultured anaerobic fungi and methanogens, as well as the problems caused by co-cultures being strictly anaerobic.

## 6. Conclusions

The main problem to address regarding the use of lignocellulosic materials for methane production is the pretreatment and hydrolysis process of recalcitrant lignocellulose. In recent years, anaerobic fungi have exhibited exceptional potential for the degradation of natural lignocellulosic materials. The activity of fibrolytic enzymes and the digestibility of lignocellulosic materials by anaerobic fungi has been shown to be significantly improved by co-culturing with methanogens, due to alteration of the metabolic pathway in fungal cells. The co-culture of isolated anaerobic fungi and methanogens has shown a tacit understanding of their interdependence and mutualism, which can improve the efficiency of methane production by replacing chemical or physical pretreatment and reducing energy consumption, thus, bringing a wide range of treatment benefits. Research on the degradation of lignocellulose and the conversion of methane by co-cultures might help to alleviate the current dependency on non-renewable fossil fuels and global energy shortages. However, more studies are needed on the selection of fermentation materials and the growth behaviors of co-cultured anaerobic fungi and methanogens, for example, the optimal proportion of methane produced by mixing lignocellulosic materials with low-value wastes and how to adapt to a wider range of temperatures and pH values to improve the efficiency of H_2_ to CH_4_ conversion. Strengthening these studies would provide new information for the development of lignocellulosic materials and their use in large-scale industrial production of methane.

## Figures and Tables

**Table 1:** The characterized morphological features and isolation source of anaerobic fungi genera.

Genus	Morphology	Isolation Source	References
*Neocallimastix*	Monocentric, Polyflagellate, Filamentous	Sheep rumen contents	[28]
*Caecomyces*	Monocentric, Uniflagellate, Bulbous	Horse caecum	[29]
*Orpinomyces*	Polycentric, Polyflagellate, Filamentous	Holstein steer rumen	[30]
*Piromyces*	Monocentric, Uniflagellate, Filamentous	Holstein steer rumen	[30]
*Anaeromyces*	Polycentric, Uniflagellate, Filamentous	Cow rumen	[31]
*Cyllamyces*	Polycentric, Uniflagellate, Bulbous	Cow feces	[32]
*Buwchfawromyces*	Monocentric, Uniflagellate, Filamentous	Buffalo feces	[33]
*Oontomyces*	Monocentric, Uniflagellate, Filamentous	Indian camel stomach	[34]
*Pecoramyces*	Monocentric, Uniflagellate, Filamentous	Sheep feces	[35]
*Feramyces*	Monocentric, Polyflagellate, Filamentous	Barbary sheep	[36]
*Liebetanzomyces*	Monocentric, Uniflagellate, Filamentous	Goats rumen samples	[37]
*Agriosomyces*	Monocentric, Uniflagellate, Filamentous	Mouflon sheep feces	[38]
*Aklioshbomyces*	Monocentric, Uniflagellate, Filamentous	White-tailed deer feces	[38]
*Capellomyces*	Monocentric, Uniflagellate, Filamentous	Boer goat feces	[38]
*Ghazallomyces*	Monocentric, Polyflagellate, Filamentous	Axis deer feces	[38]
*Joblinomyces*	Monocentric, Uniflagellate, Filamentous	Goat feces	[38]
*Khoyollomyces*	Monocentric, Uniflagellate, Filamentous	Grevy’s zebra feces	[38]
*Tahromyces*	Monocentric, Uniflagellate, Filamentous	Nilgiri tahr feces	[38]

**Table 2:** Taxonomy of methanogens.

Order	Family	Genus	Number of Valid Published Species
Methanobacteriales	Methanobacteriaceae	*Methanobacterium*	24
		*Methanobrevibacter*	15
		*Methanosphaera*	2
		*Methanothermobacter*	8
	Methanothermaceae	*Methanothermus*	2
Methanococcales	Methanocaldococcaceae	*Methanocaldococcus*	7
		*Methanotorris*	2
	Methanococcaceae	*Methanococcus*	4
		*Methanothermococcus*	2
Methanocellales	Methanocellaceae	*Methanocella*	3
Methanomicrobiales	Methanocalculaceae	*Methanocalculus*	6
	Methanocorpusculaceae	*Methanocorpusculum*	4
	Methanomicrobiaceae	*Methanoculleus*	11
		*Methanofollis*	5
		*Methanogenium*	4
		*Methanolacinia*	2
		*Methanomicrobium*	1
		*Methanoplanus*	2
	Methanoregulaceae	*Methanolinea*	2
		*Methanoregula*	2
		*Methanosphaerula*	1
	Methanospirillaceae	*Methanospirillum*	4
Methanosarcinales	Methanosarcinaceae	*Halomethanococcus*	1
		*Methanimicrococcus*	1
		*Methanococcoides*	4
		*Methanohalobium*	1
		*Methanohalophilus*	4
		*Methanolobus*	7
		*Methanomethylovorans*	3
		*Methanosalum*	2
		*Methanosarcina*	13
	Methanotrichaceae	*Methanothrix*	2
	Methermicoccaceae	*Methermicoccus*	1
Methanopyrales	Methanopyraceae	*Methanopyrus*	1
Methanomassiliicoccales	Methanomassiliicoccaceae	*Methanomassiliicoccus*	3

**Table 3:** Summary of methanogenesis in co-cultured anaerobic fungi and methanogens with different lignocellulosic materials.

Combinations of Anaerobic Fungi and Methanogens	Substrate	Incubation Time	Conversion Rate of Methane	Reference
*Neocallimastix frontalis* PN1 *+ Methanobrevibacter* sp. strain RA1	Whatman no. 1 filter paper	3 days	1.78 mmol methane/g substrate	[29]
	Whatman no. 1 filter paper	7 days	3.35 mmol methane/g substrate	[29]
	Sisal twin fiber	7 days	2.1 mmol methane/g substrate	[83]
	Barley straw leaf	7 days	1.7 mmol methane/g substrate	[83]
*Neocallimastix frontalis* ATCC 76100 + *Methanobacterium formicicum* DSM 3637	Cellulose	7 days	5.7 mmol methane/g substrate	[12]
*Neocallimastix frontalis* ATCC 76100 + *Methanosaeta concilii* DSM 6752	Cellulose	17 days	4.3 mmol methane/g substrate	[12]
*Neocallimastix frontalis* PNK2 *+ Methanobrevibacter smithii* PS	Fresh ryegrass stem	6 days	8.75 mL methane/g substrate	[99]
	Fresh ryegrass leaf	6 days	8 mL methane/g substrate	[99]
*Piromyces + Methanobrevibacter thaueri* CW	Rice straw	4 days	1.05 mmol methane/g DM	[8]
	Wheat straw	4 days	1.16 mmol methane/g DM	[8]
	Maize stem	4 days	0.71 mmol methane/g DM	[8]
	Corncob	4 days	2.17 mmol methane/g DM	[8]
	Wheat bran	4 days	1.06 mmol methane/g DM	[8]
*Piromyces + Methanobrevibacter* sp. Z8	Rice straw	4 days	1.06 mmol methane/g DM	[8]
	Wheat straw	4 days	0.96 mmol methane/g DM	[8]
	Maize stem	4 days	0.58 mmol methane/g DM	[8]
	Corncob	4 days	1.90 mmol methane/g DM	[8]
	Wheat bran	4 days	1.23 mmol methane/g DM	[8]
*Orpinomyces* sp. *+ Methanobrevibacter* sp.	Corn core	4 days	1.61 mmol methane/g substrate	[85]
*Neocallimastix* sp + *Methanobrevibacter* sp.	Corn core	4 days	1.96 mmol methane/g substrate	[85]
*Pecoramyces* sp. *+* Methanobrevibacter sp.	Corn stover leaf blade	3 days	56.6 mL/g digested substrate	[94]

## Data Availability

Data sharing not applicable.
